# Ecological niches for colorectal cancer stem cell survival and thrival

**DOI:** 10.3389/fonc.2023.1135364

**Published:** 2023-04-13

**Authors:** Jiayun Che, Shiyan Yu

**Affiliations:** ^1^ Shanghai Institute of Precision Medicine, 9^th^ Hospital affiliated to Shanghai Jiao Tong University School of Medicine, Shanghai, China; ^2^ Department of Oncology, 9^th^ Hospital affiliated to Shanghai Jiao Tong University School of Medicine, Shanghai, China

**Keywords:** colorectal cancer stem cell, immunosuppressive tone, cancer niche, colorectal cancer, oncogenic mutation

## Abstract

To date, colorectal cancer is still ranking top three cancer types severely threatening lives. According to cancer stem cell hypothesis, malignant colorectal lumps are cultivated by a set of abnormal epithelial cells with stem cell-like characteristics. These vicious stem cells are derived from intestinal epithelial stem cells or transformed by terminally differentiated epithelial cells when they accumulate an array of transforming genomic alterations. Colorectal cancer stem cells, whatever cell-of-origin, give rise to all morphologically and functionally heterogenous tumor daughter cells, conferring them with overwhelming resilience to intrinsic and extrinsic stresses. On the other hand, colorectal cancer stem cells and their daughter cells continuously participate in constructing ecological niches for their survival and thrival by communicating with adjacent stromal cells and circulating immune guardians. In this review, we first provide an overview of the normal cell-of-origin populations contributing to colorectal cancer stem cell reservoirs and the niche architecture which cancer stem cells depend on at early stage. Then we survey recent advances on how these aberrant niches are fostered by cancer stem cells and their neighbors. We also discuss recent research on how niche microenvironment affects colorectal cancer stem cell behaviors such as plasticity, metabolism, escape of immune surveillance as well as resistance to clinical therapies, therefore endowing them with competitive advantages compared to their normal partners. In the end, we explore therapeutic strategies available to target malignant stem cells.

## The theory of cancer stem cells

According to the cancer stem cell hypothesis, all cancer daughter cells emanate from self-renewal cancer stem cells (1). Although controversies exist, this theory was first demonstrated in the study of leukemia (2). The isolation and identification of cancer stem cells in solid tumors was first obtained from breast cancer with a surface marker of CD44^+^ CD24^low^ Lineage^-^ B38.1^+^ ESA^+^ (3). Subsequently, more cancer stem cells were identified in different tumor types such as brain cancer (4), prostate cancer (5), colon cancer (6) and pancreatic cancer (7). A growing number of studies implicate that cancer stem cells play an important role in tumor initiation, metastasis, drug resistance and recurrence.

## The cell of origin of colorectal cancer

**Figure 1:**
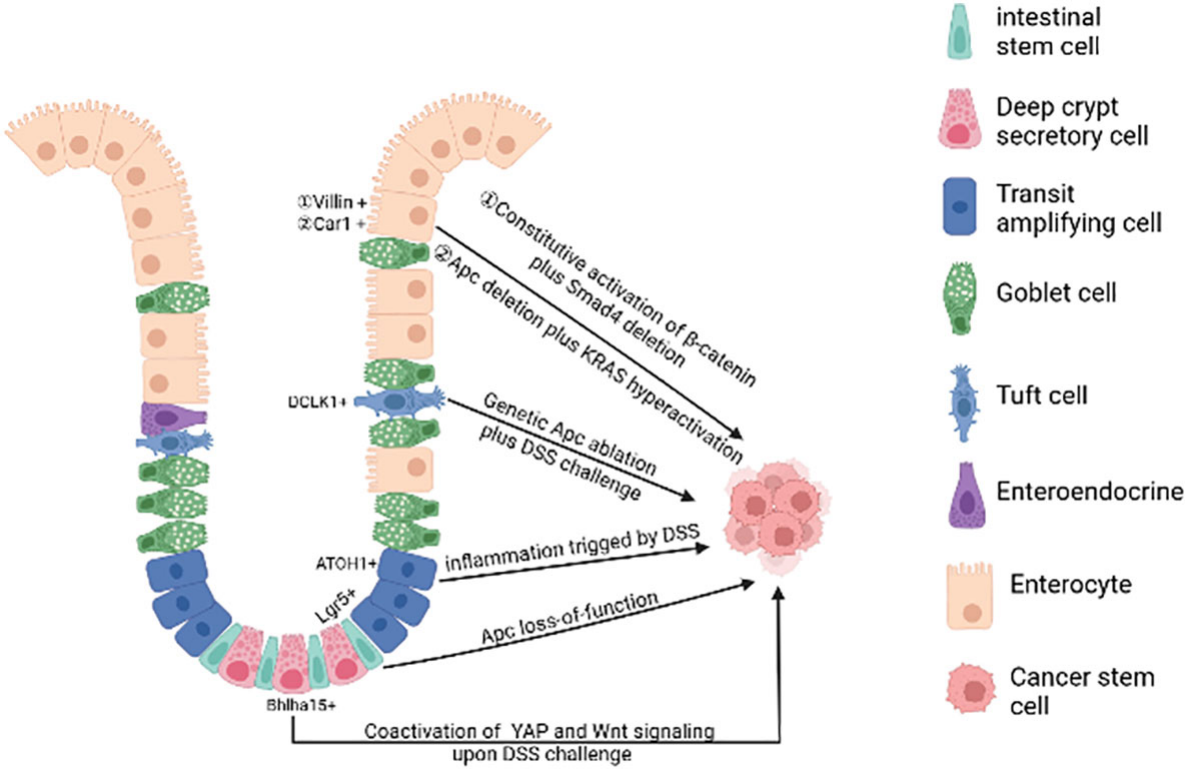
The cell of origin of colorectal cancer. A single gene mutation of the Wnt signaling pathway(such as *Apc* deletion) in intestinal stem cells prompts them to transform into cancer stem cells. Bhlha 15^+^ and ATOH1^+^ precursor cells can also transform malignantly upon DSS challenge, initiating bottom-up tumorigenesis. *Apc* mutation plus dysregulation of multiple signaling pathways (such as NFκB, SMAD, YAP) or under long-lasting inflammatory milieu may induce dedifferentiation of terminally differentiated cells (enterocytes and tuft cells), initiating top-down tumorigenesis. (Created in BioRender.com).

In physiological state, human intestinal epithelial cells have high cell turnover rate due to confrontation with constant aggressions from the lumen. In every 4-5 days, most of the epithelial cells in the intestinal tract will be replenished by new functional epithelia given rise by intestinal stem cells (ISCs). Therefore, on the one hand, the proliferation of intestinal stem cells makes it possible to accumulate gene mutations. On the other hand, rapid cell turnover of the intestinal epithelial cells also prevents the epithelial cells from accumulating mutations, leading to the hypothesis that long-lived ISCs are the most likely tumorigenesis cell. At present, the debate on the cells of origin of CRC mainly consists of two hypotheses: “bottom-up” (8) and “top-down” (9) histogenesis of colorectal tumors. The former hypothesis suggests that tumorigenesis begins in intestinal stem cells or lineage precursors in the crypt and then spreads up the crypt, the latter hypothesis suggests that differentiated cells located at the top of the crypt acquire stem-like characteristics *via* mutation, extending downwards and laterally (**Figure 1**).

A plethora of studies using genetic mouse models have illustrated that mutations in a single gene of the Wnt signaling pathway in stem cells are sufficient to form adenomas in mice. For example, targeted deletion of *Apc* in Lgr5^+^ ISC over-activates Wnt signaling, leading to rapid adenoma formation (10). The loss of *Apc* in Lrig1^+^ quiescent intestinal stem cells leads to tumors in the distal colon (11). In addition, the activation of Wnt signalling in Bmi1^+^ or CD133^+^ ISCs forms small intestinal adenomas in mice (12, 13). However, it is important to emphasize that there are some differences in tumor development in mouse models versus CRC patients, suggesting that additional factors must be taken into account when describing the origin of CRC. In mouse models of CRC with relevant genetic mutations, adenoma formation is mostly in the small intestine and rarely progresses to full cancer (14) and the development of human colorectal cancer is strongly influenced by environmental factors such as chronic inflammatory conditions (15, 16), whereas in genetic mouse models these factors are not usually included.

To recapitulate human CRC progression, targeted mutations of the most commonly mutated colorectal cancer genes such as *APC, SMAD4, P53, KRAS* and/or *PIK3CA* were introduced in cultured human intestinal stem cells using CRISPR/Cas9 technology *in vitro* (17, 18). Combination of these mutations allows human intestinal stem cells to grow in absence of stem cell niche factors, while the *APC/P53* double knockout organoids are sufficient to acquire chromosomal instability, leading to the appearance of aneuploidy, a hallmark of tumor progression (17, 18). In addition, the quadruple mutations enables human intestinal stem cells to develop xenografts with invasive carcinoma features (17, 18). The above results further confirm that human intestinal stem cells carrying oncogenic mutations can initiate oncogenesis.

Mutations in mismatch repair (MMR) genes, primarily in MutS homolog 2 (*MSH2*) and MutL homolog 1 (*MLH1*), cause microsatellite instability (MSI) and predisposition to early onset of CRC (19). To study roles of MMR deficiency in intestinal stem cell transformation, Keysselt et al. examined intestinal epithelial specific *Msh2* knockout mice. Organoids derived from *Msh2*-deficient mice exhibited inheritable transient cyst-like growth even in absence of R-spondin, suggestive of hyperactivated Wnt signaling in the stem cells. Even before detectable polyps, tumor precursor cells increased in *Msh2* ^-/-^ mice, which can form MSI-high organoids with temporary spherical shape *in vitro* similar to tumor-like organoids. Thus MMR-deficiency predisposes ISCs to oncogenic mutation accumulation and ready for transformation (20).

Additional oncogenic mutations targeted to multiple signaling pathways under repairable inflammation (21) are required to the de-differentiation of non-stem cells. These dedifferentiated cells then gain stem cell-like characteristics and initiate oncogenesis. For example, loss of *Apc* in long-lived Dclk1^+^ tuft cells is not sufficient to drive colon carcinogenesis. However, dextran sodium sulfate (DSS)-induced colitis facilitates the formation of colon cancer in these *Apc*-mutated cells (22). After DSS induction, the YAP/Wnt signaling pathway is activated, further promoting the transformation of Bhlha15^+^ secretory cell precursors into cancer-initiating cells in mouse colon (23). Similarly, in the azoxymethane-DSS CRC model, DSS triggering inflammation promotes colonic ATOH1^+^ IECs to acquire cancer stem cell-like properties thus facilitating the development of colitis-associated tumors (24).

Genetic mouse studies illustrate that constant activation of multiple pathways (e.g. Wnt and NF-κB signaling) in terminally differentiated intestinal epithelial cells can also forcefully drive them to form intestinal adenomas (25). Both *Apc* deletion and *K-ras* hyperactivation synergistically cause Car1-expressing differentiated colonic epithelial cells to obtain cancer stem cell properties and initiate top-down tumorigenesis (26). Recent studies have shown that activated Wnt signaling when accompanied by *SMAD4* deletion drives dedifferentiation and adenoma formation in differentiated intestinal epithelial cells (27). The mutation of the *Apc* along with TGFβ type 1 receptor (*Tgfbr1/Alk5*) does not lead to the adenoma formation. However, the combination of two mutations can promote dedifferentiation of intestinal epithelial cells with *Kras^G12D/+^* mutation, thus accelerating oncogenesis (28).

In addition, changes in the intestinal microenvironment can also trigger the transformation of differentiated cells. For example, the BMP antagonist GREM1 is originally expressed by mesenchymal cells at the base of the crypt to maintain a low concentration of BMP at the base of the crypt, thus maintaining the stemness of intestinal stem cells (29). But when the intestinal epithelial cells express GREM1 abnormally due to gene duplication, these cells acquire stemness and form ectopic crypts due to blockage of differentiation-promoting BMP signal (30). After accumulation of necessary somatic mutations, these ectopic crypts eventually progress to polyps (30).

## The microenvironment of colorectal CSCs

**Figure 2:**
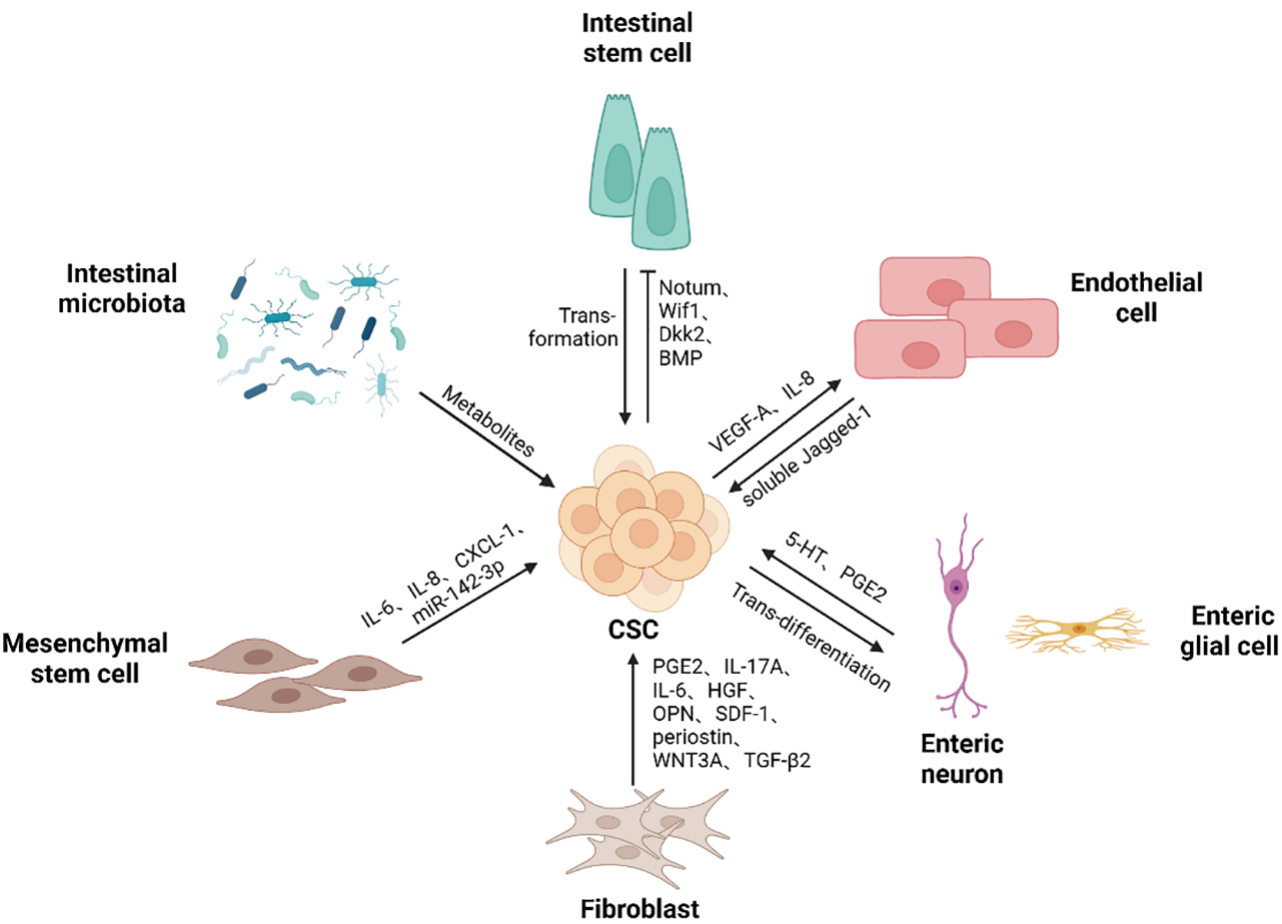
Colorectal CSCs niche components. Colorectal CSCs niche contains different types of cells and microbes, as well as the soluble substances they produce, supporting the self-renewal ability of cancer stem cells. Cancer stem cells can gain a competitive advantage over normal stem cells by promoting differentiation. Mutations drive intestinal stem cells to transform into cancer stem cells. Besides, colorectal cancer stem cells promote endothelial cell proliferation and migration, thereby participating in the generation of blood vessels. In addition, cancer stem cells give rise to neurons, thus promoting tumor development. (Created in BioRender.com).

To maintain colorectal CSCs in CRC, various cell types and even microbes are recruited or reprogramed to cooperate within CSCs to construct ecological niches. Within these niches, colorectal CSCs can utilize a variety of resources to self-renew and give rise to all malignant daughter cells while avoiding immune attacks (**Figure 2**).

### Colorectal CSCs outcompetition of wildtype stem cells

Studies have demonstrated that cancer stem cells gain a competitive advantage over normal stem cells to achieve the spread of mutant clones. Both *Apc* loss and *K-Ras* activation in ISCs endorse them a clonal advantage over surrounding stem cells in the mouse intestine, while *P53* mutations endow a condition-dependent advantage, especially in colitis-associated colon cancer (31, 32). In addition, recent studies have reported that stem cells carrying pro-tumor mutations produce multiple factors that affect the proliferation and differentiation of neighboring stem cells, thus increasing the crypt fixation of mutated stem cells by reducing the number of normal stem cells at the base of the crypt and promoting the initiation of cancer (33). Yum et al. reported that intestinal stem cells containing oncogenic mutations (e.g. *Apc, KRAS* and *PI3K*) could promote the differentiation of normal stem cells by secreting factors that activate the BMP signaling pathway and inhibit the WNT signaling pathway. Similarly, PI3K mutant stem cells induced the secretion of the WNT inhibitor BMP by nearby PDGFR^low^ CD81^+^ stromal cells, which together affect Wnt signaling in the microenvironment (34). Van Neerven et al. discovered that *Apc* mutant ISCs outcompeted neighboring wildtype stem cells by secreting WNT antagonists (Notum, Wif1 and Dkk2), forcefully triggering wildtype undergo differentiation. (35). Lithium chloride, GSK3β inhibitor, treatment could prevent this outcompetitive effect in wildtype stem cells, thereby making wild-type ISCs insensitive to WNT antagonists and preventing adenoma formation (35). In consistence, Flanagan et al. also described that the expression of Notum was highest among multiple secretory WNT antagonists in *Apc* mutant cells. Inhibition of NOTUM impaired the ability of mutant cells to expand and form intestinal adenomas, suggesting that targeting NOTUM can restore the competitiveness of wild-type cells (36).

### The interactions between colorectal CSCs and endothelial cells

In solid tumors, vasculature is an essential component of the microenvironment of cancer stem cells, which provides necessary nutrients, oxygen and other substances for the growth of solid tumors, while taking away tumor metabolites. It is also the main pathway for tumor to communicate with the tissues and organs of the whole body. In human colorectal cancer, cancer stem cells were found to be always present near tumor vessels (37, 38), indicating a close relationship between CSCs and tumor vessels. Three main hypotheses are proposed for tumor angiogenesis. one hypothesis is that tumors form blood vessels by inducing the sprouting, proliferation and migration of the existing vessels. Studies on stem cell-like glioma cells have revealed that CSCs can secrete vascular endothelial growth factor (VEGF) and stromal-derived factor 1 (SDF1) to promote endothelial cell proliferation, migration and tubular structure formation (39, 40). A recent study found that GATA6 transformed stem-like HCT-116 and HT-29 cells could promote the migration, proliferation, invasion and tube formation of human umbilical vein endothelial cells (HUVECs) *in vitro* by elevated secretion of IL-8 and VEGF-A due to the EGFR/AKT-mediated activation of NF-κB (41). The second hypothesis suggests that tumor recruits bone-marrow-derived endothelial precursor cells (EPCs) to differentiate into endothelial cells that participate in neovascularization (42). Wei et al. found that colon cancer stem cells promoted proliferation, migration and tube formation of EPCs by secreting vascular endothelial growth factor (VEGF) *in vitro*, suggesting that colorectal CSCs are more likely to promote tumor neovascularization by recruiting EPCs *in vivo*. Meanwhile, tumorigenic assays in nude mice showed that EPCs increased the tumorigenic and metastatic capacity of CSCs through vasculogenesis (37). The third hypothesis tumor vasculogenesis is that cancer stem cells can differentiate into vascular endothelial cells (43–45) and pericytes (46) that make up and function as blood vessels in tumors. A recent study detected blood endothelial cells expressing human cell-specific nuclear antigen NuMA, CD31, and VEGFR2 in xenografts derived by colorectal CSCs, indicating that CSCs are capable of generating vascular endothelial cells and constitute functional blood vessels in cancer tissues (47).

Vascular endothelial cells not only play a supportive role in vessels, but also influence the CSC phenotype by secreting soluble factors. Dr. Ellis lab uncovered that vascular endothelial cells could release soluble Jagged-1 *via* ADAM17 cleavage to activate Notch signaling in CD133^+^ colorectal CSCs to maintain cancer stem cell phenotype (38). Recently the same group also reported that endothelial cells secrete soluble factors activating cancer cell HER3/AKT signaling to promote these cells survival (48).

### The interactions between CSCs and nervous system

The role of the enteric nervous system (ENS) in promoting the growth and metastasis of colorectal cancer has been reviewed (49, 50). Perineural invasion (PNI) and neoneurogenesis are considered to be the two major factors that play a role in CRC. PNI is defined as tumor invasion of nerve structures and spread along the nerve. The severity of PNI is associated with poorer survival and has been identified as an independent prognostic factor of outcomes (51). Cancer cells induce neurogenesis in tumors by secreting signaling molecules and neurotrophic factors (52–54). Lu et al. found that human colorectal cancer stem cells could guide the construction of ganglia in and around tumor masses when forming xenograft tumors in the peritoneal cavity of immunodeficient nude mice (55). In addition, these cancer stem cells gave rise to neurons with synaptic markers as well as with sympathetic and parasympathetic neuronal markers *in vitro* (55). Disruption of CSCs neural differentiation potential inhibited the growth of their transplanted tumors in immunodeficient animals (55).

Enteric nerve cells and enteric glial cells, which make up the enteric nervous system, can influence colorectal cancer development and metastasis by regulating the activity of CSCs. Tph2 critical for 5-hydroxytryptamine (5-HT) biosynthesis is increased in CRC tissues. Zhu et al. have demonstrated that enteric serotonergic neurons secrete 5-HT to promotes CSC self-renewal and tumorigenesis. Mechanistically, 5-HT receptor HTR1B/1D/1F is highly expressed on the surface of colorectal CSCs. Once binding to its receptor, 5-HT will license the interaction of HTR with AXIN1 protein and affect the membrane translocation of AXIN1 protein, which further activate the Wnt/β-catenin signaling by inhibiting the assembly of the β-catenin degradation complex (56). Screening of factors inducing Tph2 expression leads to identification of isovaleric acid from gut microbiota metabolome. Mechanistic studies further reveal that isovaleric acid can interrupt inhibitory NuRD transcriptional complex to dock on Tph2 promoter, thus initiating Tph2 expression (56). Enteric glial cells (EGCs) produce more PGE2 induced by IL-1α/β derived from malignant epithelial cells. The production of PGE2 can enhance the tumorigenicity and expansion of CSCs *via* the Ep4/EGFR/ERK1 pathway. It is also suggested that chronic inflammatory stress or elevated local cytokine levels contributes to the phenotypic remodeling of EGCs, which may be an early event in colon carcinogenesis and promote CSC-derived tumor formation (57).

### The interactions between CSCs and cancer-associated fibroblasts

In solid tumors, CAFs are the most indispensable stromal cells (58). Single cell RNA profiling has revealed that CAFs contain functionally heterogenous subpopulations (59–61). CAFs deposit a large amount of extracellular matrix protein (ECM) such as collagen and fibronectin involved in construction of tumor architecture. The dense ECM can inhibit the penetration of immune cells and even anti-tumor drugs. CAFs also secret matrix metallopeptidases to continuously remodel tumor mass, promoting cancer cell invasion. In addition, CAFs secret various cytokines involved in angiogenesis, immune evasion, and CSC maintenance (62, 63).

Roulis et al. have identified a population of pericryptal Ptgs2-expressing fibroblasts by single-cell RNA-Seq. These Ptgs2 expressing fibroblasts can process arachidonic acid into highly labile prostaglandin E2 (PGE2), which induces dephosphorylation and nuclear translocation of Hippo pathway effector Yap through the receptor Ptger4-mediated signaling pathway, therefore promoting the proliferation of Sca-1^+^ reserve stem cells and driving tumor initiation (64). In human colon cancer, a significant increase in the number of IL-17A expressing CAFs after chemotherapy promote the self-renewal ability, resistance to chemotherapy and invasiveness of CD44^high/+^ cancer stem cells (65). Additionally, CAFs isolated from human CRC tumors can secrete hepatocyte growth factor (HGF), osteopontin (OPN) and SDF-1 to increase CD44v6 expression in colorectal CSCs, thereby initiating tumor migration and metastasis (66). The expression of CD44v6 is not only related to tumor metastasis, but also to drug resistance of cancer stem cells. After FOLFOX chemotherapy, CAFs secrete factors such as periostin, IL-17A and WNT3A to activate the WNT3A/β-catenin signaling pathway, which not only leads to the persistent tumorigenic ability of CSCs, but also induces the expression of CD44v6 in CSCs and promotes the resistance of CSCs to FOLFOX (67). In the hypoxic tumor microenvironment, TGF-β2 derived from CAFs cooperates with hypoxia-induced production of HIF-1α to induce the expression of hedgehog transcription factor GLI2 in CSCs, promoting the self-renewal ability and robust resistance to chemotherapy in CSCs (68).

Additionally, CAFs can promote cancer progression by affecting the plasticity of cancer cells. In T2-T3 stage CRC, CD90^+^ CAFs are the main source of IL-6 in the tumor microenvironment and can promote the expression of stem cell markers ALDH and Lgr5 in cancer cells, thereby promoting cancer development (69). CAFs can also promote the stem cell-like characteristics of CRC cells by transferring exosomal lncRNA H19 to colorectal cancer cells, which activates the expression of β-catenin *via* acting as a competing endogenous RNA sponge for miR-141 (70).

Of note, studies have reported that a subpopulation of CAFs can function as tumor suppressors (63, 71, 72). McAndrews et al. demonstrated that in αSMA^+^ CAF-depleted tumors, the expression of CSC markers Lgr5, CD44, DCLK1 increased in comparison to control group. Further analyses showed that αSMA^+^ CAFs in colorectal cancer inhibited the proliferation of Lgr5^+^ CSCs and promoted the differentiation of CSCs through BMP4/TGFβ1 signaling pathway, exerting tumor suppressive effects and inhibiting the CRC progression (73).

### The interactions between CSCs and mesenchymal stem cells

MSCs belong to the pluripotent stem cells and have multiple differentiation potentials to give rise to many lineages, such as adipocytes, osteocytes and chondrocytes (74). They can be recruited to the site of tissue injury or inflammation through endocrine signaling and perform tissue repair functions (75, 76). Cancer is also known as the wound that never heals (77). It has been proved that MSCs can be recruited to the tumor sites and transform into tumor-­associated MSCs (TA­MSCs) or differentiate into CAFs to affect the progression of the cancer (78, 79).

In colorectal cancer, CD133^+^/CD44^+^ colon cancer stem cells have a stronger capacity of bone marrow derived mesenchymal stem cells (BM-MSCs) recruitment compared with CD133^-^/CD44^-^ colon cancer cells due to IL-8/CXCR2 chemotaxis (80). Recruited MSCs are involved in maintaining and promoting CSC stemness. MSCs can secrete IL-6 to promote stem cell marker CD133 expression in colorectal cancer cells through activation of the JAK-STAT3 signaling pathway (81). Similarly, MSC-like cells isolated from human colon cancer can also secrete IL-6 and enhance the expression of stem cell marker CD44 in HCT-116 and HT-29 cells through the Notch signaling pathway (82). In addition, IL-1 derived from cancer cells induces PGE2 secretion of BM-MSCs. The resulting PGE2 cooperating with IL-1 paracrine signaling further promotes BM-MSCs to release IL-6, IL-8 and GRO-α, causing the accumulation of β-catenin in cancer cells and the formation of ALDH^high^ CSCs associated with increased capacity for cancer cell metastasis and invasion. (83). Jiménez et al. have also demonstrated that conditioned medium from human mesenchymal stem cells (CM-MSCs) enriches and maintains a subpopulation of colonic cells with the expression of CSC markers and ALDH activity (84). BM-MSCs also interact with cancer stem cells through exosomes. A recent study has shown that miR-142-3p in exosomes derived from BM-MSCs increased the number of CSCs in colon cancer *via* activation of Notch signaling by interfering with Numb target genes (85).

## Colorectal cancer immunogenicity affects immune response types

CRCs with MSI-high and microsatellite stable (MSS) have different tumor microenvironments. The density of CD8^+^ cytotoxic T cells in tumor glands is significantly increased in MSI-high patients compared to MSS (86–91). Similarly, Th1 cells are generally enriched in MSI-high CRCs (88–90). Two IFNG^+^ Th1-like cell clusters are identified and only CXCL13^+^BHLHE40^+^ Th1-like cells were preferentially rich in patients with MSI-high tumors (92). In contrast, Th17 infiltration is significantly increased in patients with MSS tumors (89). In MSI-high CRC tissues, more CD20^+^ B cells occur in the tumor’s invasive margin (IM) compared to MSS tumors (89). Using the CIBERSORT algorithm, Lin et al. further demonstrate that most anti-tumor immune cells such as CD8^+^ T cells, activated memory CD4^+^ T cells, follicular T helper cells, NK cells, M1 macrophages and neutrophils cells increase in the tumor microenvironment of MSI-high CRCs, but Treg cells significantly decrease. By contrast, in MSS/MSI-L CRC, Treg cells significantly upregulate, suppressing the killing function of T cells (90).

### Tumor-associated macrophages

Tumor-associated macrophages (TAMs) are active infiltrative inflammatory cells in the tumor microenvironment (93, 94). In colorectal cancer, TAMs are mainly derived from monocytes, which arise from bone marrow or tissue resident-derived precursors and monocytic myeloid-derived suppressor cells (M-MDSC). The roles of TAMs in colorectal cancer have been well reviewed elsewhere (95, 96). The specific interactions between CSCs and TAMs have been explored. For example, colonic CSCs induce milk-fat globule-epidermal growth factor-VIII (MFG-E8) expression in TAMs, which in turn promotes tumorgenicity and chemoresistance of CSCs by STAT3 and Sonic Hedgehog signaling pathways (97). Chemotherapy-resistant cancer stem cells (CSCs-R) tend to secret more pro-inflammatory substrates, shaping the tumor microenvironment different from untreated cancer stem cells. CSCs-R promote macrophage colony-stimulating factor (M-CSF) production *via* interferon regulatory transcription factor 5 (IRF5) dependent manner, which induce CD14^+^ monocytes to differentiate into M2 TAMs (98).

Reciprocally, TAMs facilitate differentiated tumor cells to acquire CSCs-like characteristics through secreting various mediators contributing to tumor proliferation and metastasis. The secretion of IL-6 by transformed M2 TAMs promotes cancer cells to express more YAP1, K-Ras, β-catenin, NF-κB, and mTOR and thus enhances the percentage of cancer stem-like cells (99). TAM can also regulate the stemness of tumor cells by affecting metabolism. TAMs isolated from colorectal cancer patients secrete transforming growth factor-β (TGF-β), promoting cancer cell glycolysis. Glycolysis activates the HIF1α/Tribbles pseudokinase 3 (TRIB3) signaling pathway, leading to activation of the β-catenin/Wnt signaling pathway and ultimately enhancing stem cell-like phenotype and cell invasion in colorectal cancer (100).

### Myeloid-derived suppressor cells

MDSCs are a heterogeneous class of myeloid cells composed of two main subgroups: polymorphonuclear myeloid-derived suppressor cells (PMN-MDSCs) and monocytic myeloid-derived suppressor cells (M-MDSCs) (101, 102). In most cancer types, PMN-MDSCs account for more than 80% of all MDSCs (103) and have a closely relationship with tumor-associated neutrophils (TAN) in the tumor environment (104, 105). M-MDSCs recruited to the tumor can differentiate into TAMs (106).

MDSCs in colorectal cancer can inhibit cytotoxicity of CD8^+^ T cells and NK cells by secreting TGF-β, NO and ROS, interrupt B cells from producing antibodies against tumor-associated antigens (TAA), induce Treg cells, and exert immunosuppressive effects in other ways (107). CXCR2-positive MDSCs are recruited into the tumor microenvironment *via* chemokines CXCL1 and CXCL2 mainly expressed in tumor colonic epithelial cells, which is critical for colitis-associated tumor formation and progression (108). PMN-MDSCs promote the stemness of CSCs in colorectal cancer *via* exosomal delivery of protein S100A9. S100A9 can activate NF-κB and STAT signaling pathways, thus affecting colorectal carcinogenesis and recurrence. Notably, these influences are amplified by HIF-1α under hypoxic conditions (109).

### Dendritic cells

Dendritic cells (DCs) are one of critical anti-tumor cell types by presenting processed neoantigens to T cells and thus facilitating activation and expansion of antigen specific T cells. However, in advanced CRC tissues DCs are castrated. Colorectal cancer stem cells can perform immune escape by inhibiting the antigen presentation ability of DCs. Zhong et al. show that TGF-β1 derived from spheres of high-stemness colorectal cancer cell lines CMT93 and CT26 down-regulates the surface expression of major histocompatibility complex Class II of bone marrow derived DCs (BMDCs) and inhibits the stimulation of T cells by BMDCs (110). In addition, CSCs can sabotage DC activation. The more CD133^+^ CSCs are present in the tumor samples, the less DCs are activated after stimulation (111). Meanwhile, tolerogenic DCs in the tumor microenvironment also enhance the stemness of cancer cells. For example, CXCL1 secreted by tumor-associated dendritic cells (TADCs) increases the CSCs of colon cancer by promoting CD133 expression and acetaldehyde dehydrogenase activity (112).

### Regulatory T cells

Regulatory T cells are widely accepted as a subset of cells that exert immunosuppressive properties through multiple inhibitory mechanisms, particularly *via* secreting IL-10 and TGF-β to inhibit T cell response (113–115). Kryczek et al. also have revealed that IL-22 derived from CD4^+^ T cells could increase the stemness and tumorigenic potential of colorectal cancer cells by activation of STAT3 and expression of the histone 3 lysine 79 (H3K79) methyltransferase DOT1L, which correlates with the induction of the core stem cell genes NANOG, SOX2 and Pou5F1 (116). CSCs can recruit and activate Treg cells through various factors in several types of cancer such as melanoma (117) and breast cancer (118). In addition, tumor-infiltrating Tregs can arise from circulating naive CD4^+^ T cells that differentiate into Treg cells in cancer tissues (119). Tumor associated Treg cells promote the dedifferentiation of non-CSCs *via* TGF-β, thereby resulting in an increasing number of CD44^+^ cancer stem cells in CRC tissue (120). However, another report suggests that αSMA^+^ CAFs promote the differentiation of colorectal cancer stem cells through the BMP4/TGFβ1 signaling pathway, thereby inhibiting Lgr5^+^ CSCs (73). Therefore, the effect of TGF-β from different sources on cancer stem cells seems controversial. More studies will be needed to verify the effect of TGF-β on CRC CSCs.

### Cytotoxic NK and CD8^+^ T cells

Compared to colorectal non-CSCs, CSCs are more sensitive to the killing function of NK cells because of their high expression of ligands for natural cytotoxicity receptor (especially NKp30 and NKp44) and low expression of MHC class I molecules (121). However, the complex formed by PCNA and HLA-I interactions on the extracellular surface of tumor cells is identified as an inhibitory ligand for NKp44, thus preventing activation of cytotoxic NK cells (122). PCNA^+^ colorectal cancer cells (HCT 116) exhibit the higher expression of CSC markers CD44 and CD133 and stemness genes such as NANOG, SOX-2 and Oct-4 (123). Thus, colorectal CSCs displaying PCNA with MHC-I on their surfaces may present a possible strategy to evade the killing effect of NK cells.

CD8^+^ cytotoxic T cells are more specific anti-tumor killers. However, CD133^+^CD44^+^ colorectal CSCs express high levels of immune regulatory molecules such as PD-L1 (124), equipping CSCs to demolish cytotoxic cell activities. In addition, the high level of IL-4 is present on the membrane of colorectal CSCs, which will activate IL-4 signaling in T cells through cell-to-cell contact and inhibit T cell proliferation and cytotoxicity (125). Moreover, IL-4 also inhibits the anti-tumor activity of TH1 cells (125) while stimulates TH2 response for tissue repair, further promoting tumorigenesis.

## The roles of gut microbiome in CSCs

The critical roles of gut microbiome in the initiation, progression, metastasis and immune escape of digestive cancers have been well reviewed elsewhere (126–128). Here, we briefly focused on the effect of microbiome on colon stem cells. Various microbes in gut microbiome can promote dedifferentiation and reprogramming of intestinal epithelial cells, thereby contributing to CSCs formation (129). Previous studies have identified that genotoxic *pks+ Escherichia coli*, enterotoxigenic *Bacteroides fragilis*, *Streptococcus gallolyticus* and *Fusobacterium nucleatum* as pro-tumorigenic microbes. Recent mechanistic studies further reveal a variety of approaches by which these microbes induce CSCs. For example, genotoxic *pks+ Escherichia coli* can synthesize colibactin, alkylating genomic DNA on adenine residues in intestinal epithelial cells and therefore accelerating mutagenesis and transformation (130). Enterotoxigenic *Bacteroides fragilis* promotes CSCs by increasing histone demethylase JMJD2B (131). *Fusobacterium nucleatum* can stimulate fatty acid oxidation for CSC self-renewal and proliferation. In addition, this microorganism can also license Notch activation in non-CSCs, thus facilitating the acquisition of stem cell characteristics (132). Moreover, Ternes et al. have revealed that formats, one of *Fusobacterium* metabolites, could activate aryl hydrocarbon receptor signaling and therefore increase the expression of stem cell markers ALDH, CD44, and OCT4 (133).

## Tumor microenvironment determines immunotherapy outcome

**Figure 3:**
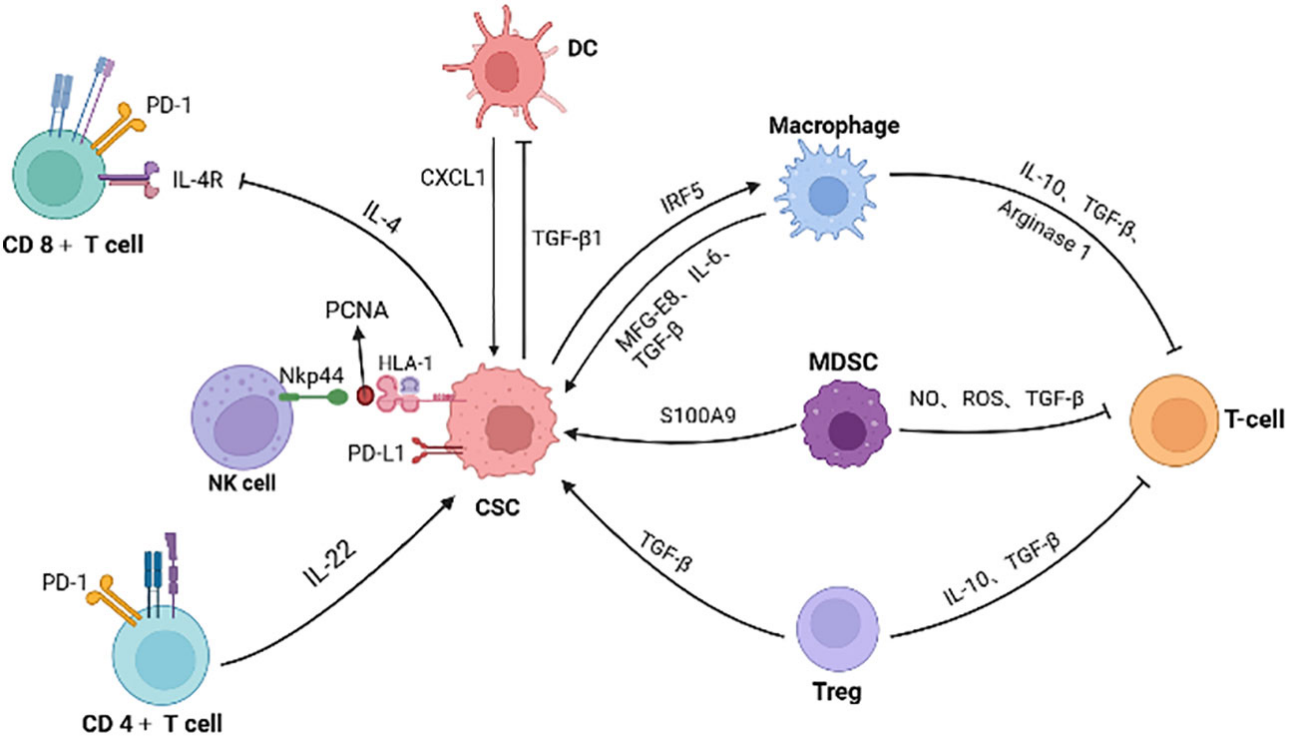
The interactions of CSC with immune cells and inhibitory effect on T cells. Immune cells (such as macrophage, MDSC, Treg and CD4^+^ T cells) secret factors or exosomes to promote stemness and increase the number of CSCs. CSCs also inhibit the function of immune cells. For example, colorectal CSCs secret IL-4 to inhibit T cell proliferation and TGF-β1 to reduce the antigens presentation of DC cells. PCNA and HLA-I interactions on the surface of CSCs is identified as an inhibitory ligand for NKp44 on NK cells, thus preventing cytotoxic activation. Meanwhile, immunosuppressive cells in the microenvironment inhibit T cells, promoting the immune escape of CSCs. (Created in BioRender.com).

As previously mentioned, CRC CSCs recruit and educate immunosuppressive neighbor cells, collectively constructing immunotolerant ecological community favoring tumor cell growth and immunotherapy resistance (**Figure 3**). For example, CAFs are associated with poorer efficacy of T-cell-based immunotherapies for CRC (134). Patients with low risk scores for CAFs have a greater response to PD-L1 inhibitors and significant clinical benefit (135). CAFs secret WNT2, which inhibits DC-mediated anti-tumor T-cell responses *via* the SOCS3/p-JAK2/p-STAT3 signaling cascade. Anti-WNT2 monoclonal antibody significantly restores anti-tumor T-cell responses and enhances anti-PD-1 efficacy by increasing active DCs (136). Similarly, MDSCs mediate tumor immune evasion and resistance to immune checkpoint inhibitors (137). The efficacy of immune checkpoint inhibitors is enhanced when the number of MDSCs is reduced (138).

Immunosuppressive cells in CRC tissues can synergistically create immune exclusion microenvironment, hindering immunotherapy. Qi et al. reported that tumor-specific FAP^+^ fibroblasts and SPP1^+^ macrophages cooperatively constructed immune-excluded desmoplasic structure and curtailed T cell infiltration. Patients with high FAP or SPP1 expression gained less therapeutic benefit from treatment against PD-1 (139). Likewise, Gyori et al. found that CSF1R^+^ TAMs and Foxp3^+^ Treg cells were the main compensatory cellular components of the immunosuppressive niches (140). When one cell type is selectively ablated, another cell type will increase compensatively (140), indicative of elasticity of CSC niches. Thus, interruption of key connections among CSCs and accessory cells within immunosuppressive ecosystem may provide new direction for cancer therapy.

In addition to key immunosuppressive cells and molecules, intestinal microbes in CRC microenvironment can actively influence the efficacy of immunotherapy. The roles of microbiome in cancer progression, immunosurveillance and therapy have been well reviewed elsewhere (141–143). To date more approaches by which the gut microbiome affects chemoradiotherapy have been revealed. For example, Bacteroides vulgates can mediate nucleotide synthesis and promote the DNA repair of tumor cells, thus reducing the efficacy of chemoradiotherapy (144).

## Effect of radiotherapy and chemoradiotherapy on tumor microenvironment

Radiotherapy can not only kill tumor cells, but also induce immune cells and reshape the tumor immune microenvironment in various ways (145, 146). One of benefits radiotherapy reverses the immunosuppressive tumor microenvironment is the abscopal effect. This effect is described as the clinical phenotype that radiotherapy at one site may lead to regression of metastatic cancer at distant sites beyond the radiotherapeutic field. The underlying mechanism is that irradiated tumor cells release neoantigens (tumor-associated antigens, TAAs) (147) and cytokines (148), altering microenvironment immune tone and induce tumor-specific immune responses to eliminate primary and metastatic tumors. A systematic review on pan-cancer demonstrates that radiotherapy leads to the increased CD3^+^ or CD8^+^ lymphocyte density and increased PD-L1 expression (149). several studies have also reported that both tumor-associated fibroblasts (150) and macrophages (151) with immunosuppressive effects are activated after radiotherapy.

Recently, chemoradiotherapy is recommended for locally advanced rectal cancer, which is beneficial for improving cure rates and maintaining function (152). A recent report comprehensively evaluates the dynamic changes of the tumor immune microenvironment in patients receiving chemoradiotherapy (153). In this study, they found that chemoradiotherapy significantly increased the density of CD3^+^ T cells, CD8^+^ T cells and dendritic cells, while decreased the density of CD4^+^FoxP3^+^ regulatory T cells, indicating that chemoradiotherapy promotes a more immune-active TIME. On the other hand, they found that chemoradiotherapy also induced immunosuppressive effect by polarizing tumor-associated macrophages from pro-inflammatory M1 macrophages to immunosuppressive M2 macrophages and reducing B cell density. Therefore, in order to reduce the immunosuppressive effect, the radiotherapy or chemoradiotherapy treatment combined with inhibition of immunosuppressive molecules may bring more benefits for patients. For example, Ji et al. verified that radiotherapy combined with anti-CD25/CTLA4 monoclonal antibody could reduce Tregs, PD1^+^CD8^+^ and PD1^+^CD4^+^ T cells and effectively mount the anti-tumor response, inhibiting the growth of local and distal unirradiated tumors (154). Besides, the combination of radiotherapy and immunotherapy also boosts the abscopal effect (155). A recent report has demonstrated that radiotherapy combined with immune cytokine L19-IL2 resulted in 75% tumor remission and 20% abscopal effect in a colon cancer cell model with T-cell inflammation, highlighting that the proper combination of radiotherapy and immunotherapy can transform CRC ecological landscape from immune resistant into immune responsive (156).

## Conclusions and perspective

CRC still ranks on top of life-threatening disease worldwide. Early endoscopic detection and removal of polyps has significantly decrease CRC-related death. However, it is a huge challenge for managing CRC of middle and advanced stages. Colorectal CSCs arise from normal stem cells, lineage precursors and even differentiated cells when they receive enough oncogenic modifications on the genome. These genetic modifications not only bring CSCs into unrestrained cell cycles, but also open windows for CSCs to continuously evolve and generate heterogeneous populations of malignant progenies. Under pressures, the most fit clones will be selected and gradually cooperate with various cell types to establish immunosuppressive niches for CSC survival and thrival. Unfortunately, majority of CRC patients did not benefit from immune checkpoint inhibitors, suggesting that these immunosuppressive niches are refractory to be disrupted. Advancement of single cell and spatial multiomics and algorithms is accelerating studies to untangle the complex communication networks within niches and identify keystone interactions. Disruption of the keystone interactions between CSCs and niche accessary cells may provide novel strategies for ICI therapy resistant patients.

## Author contributions

JC and SY designed, wrote and revised the draft. Both authors contributed to the article and approved the submitted version.
